# Engraftment of Mouse Embryonic Stem Cells Differentiated by Default Leads to Neuroprotection, Behaviour Revival and Astrogliosis in Parkinsonian Rats

**DOI:** 10.1371/journal.pone.0072501

**Published:** 2013-09-12

**Authors:** Debasmita Tripathy, Reena Haobam, Ranju Nair, Kochupurackal P. Mohanakumar

**Affiliations:** Division of Cell Biology and Physiology, Laboratory of Clinical and Experimental Neuroscience, CSIR-Indian Institute of Chemical Biology, Jadavpur, Kolkata, India; The Mental Health Research Institute of Victoria, The University of Melbourne, Australia

## Abstract

We report here protection against rotenone-induced behavioural dysfunction, striatal dopamine depletion and nigral neuronal loss, following intra-striatal transplantation of neurons differentiated from murine embryonic stem cells (mES). mES maintained in serum free medium exhibited increase in neuronal, and decrease in stem cell markers by 7th and 10th days as revealed by RT-PCR and immunoblot analyses. Tyrosine hydroxylase, NURR1, PITX3, LMX1b and c-RET mRNA showed a significant higher expression in differentiated cells than in mES. Dopamine level was increased by 3-fold on 10th day as compared to 7 days differentiated cells. Severity of rotenone-induced striatal dopamine loss was attenuated, and amphetamine-induced unilateral rotations were significantly reduced in animals transplanted with 7 days differentiated cells, but not in animals that received undifferentiated ES transplant. However, the ratio of contralateral to ipsilateral swings in elevated body swing test was significantly reduced in both the transplanted groups, as compared to control. Striatal grafts exhibited the presence of tyrosine hydroxylase positive cells, and the percentage of dopaminergic neurons in the substantia nigra was also found to be higher in the ipsilateral side of 7 days and mES grafted animals. Increased expression of CD11b and IBA-1, suggested a significant contribution of these microglia-derived factors in controlling the limited survival of the grafted cells. Astrocytosis in the grafted striatum, and significant increase in the levels of glial cell line derived neurotrophic factor may have contributed to the recovery observed in the hemiparkinsonian rats following transplantation.

## Introduction

Parkinson's disease (PD) is characterised by loss of dopaminergic neurons in substantia nigra pars compacta (SNpc) region of the brain. PD symptoms, majority being motor incoordination, result from severe loss of dopamine (DA) levels in nucleus caudate-putamen, commonly referred to as the striatum. Supplementation of DA by the administration of its precursor L-3,4-dihydroxyphenylalanine, direct activation of dopamine receptor by agonists, or by augmentation of remaining dopaminergic neurotransmission through inhibition of dopamine degrading enzymes are the most popular choices of therapies for the disease. However, with time these treatments lose their efficacy and patients develop fluctuations in motor functions, on-off phenomenon and dyskinesias. These limitations have encouraged a search for unconventional treatment paradigms, especially cell transplantation strategies, with an idea to restore or replace dopaminergic neurons in the brain.

In animal experiments it is well documented that cells transplanted into the brain regions can survive to establish connections with the host cells [1], [2], [3], however the advancement in research in the area is clearly too preliminary to take it towards clinical practice. The approach requires standardization of neuronal differentiation protocols. Further information on recovery following transplant of differentiated, differentiating, or mixed population of neural cells, and the graft survivability in the host is also warranted. Major problems envisaged are poor availability of pure, DA rich autologous cultures, lack of standardized cell culture techniques that refrain from cross species contamination, consistency in the quality of cells generated, and above all unavailability of a rich source of cells that can be differentiated into DA-rgic neurons. Moreover, the transplantation outcome and the degree of symptomatic relief in clinical trials have been controversial [2]. Previous studies, including clinical trials, performed using embryonic ventral mesencephalic tissue, not only showed considerable symptomatic recovery, but also survival of grafted cells and extensive re-innervation into the host tissue [1], [4]. But ethical concerns of using aborted foetus and the limited supply of tissue are certain problems associated with the use of ventral mesencephalic tissue. Embryonic stem cells (ES), neural stem cells and human umbilical cord blood derived mesenchymal stem cells are other sources that can generate DA neurons, and their easy availability makes them a good resource for transplantation therapy [5], [6]. The non-mesencephalic cells are primed epigenetically to differentiate towards non DA-rgic neuronal fate [5], [7]. Neural stem cells need to be reverted back to the pluripotent state before they can be induced to give a sizeable number of DA neurons [8], since these have limited renewal capacity as compared to ES [9]. Therefore, ES scores over other cell types as a suitable source for cell transplantation therapy.

There are several studies that show the generation of neurons from ES [10], [11]. These are grown and maintained on a feeder layer of mouse embryonic fibroblasts in a medium containing foetal bovine/calf serum, which may lead to unexpected viral infection and/or cross-species contamination. Thus, for human therapeutic applications, ES must be grown in a safe synthetic medium without factors or cells from other animals [12]. Owing to the above mentioned reasons we have used ES as the donor cells and differentiated them under serum free condition, in a synthetic medium and without any exogenous factors by the process of default neurogenesis [13]. These are characterized for their neural progenitor status, ability to differentiate into midbrain DA-rgic neurons and the mixed population of cells are transplanted into the striatum of a rotenone-induced hemiparkinsonian model. The present study describes the characteristics of the differentiated neurons and the host response to achieve transplantation recovery, and the molecular biomarkers that help in the process.

## Materials and Methods

### Cell maintenance and differentiation

D3 murine ES line was purchased from ATCC (No. CRL11632). The cells were maintained in DMEM supplemented with 15% foetal bovine serum (FBS), 2 mM glutamine, and 1000 Units of non-essential amino acids, 100 Units of penicillin and 100 µg streptomycin, 0.7% β-mercaptoethanol and 1000 Units of leukaemia inhibitory factor (Millipore, USA). For differentiation, specific number of cells were plated in culture flasks in DMEM (Gibco) supplemented with 10% FBS (Hyclone), other constituents being the same as in routine culture. After 48 h medium was changed to knockout DMEM supplemented with 10% knockout serum replacement along with all the other components. Cells were maintained for two different time points post-plating; 7 days and 10 days, and at these two stages the cells were characterized. Based on the results obtained only 7 days differentiated cells were used for transplantation studies.

### Animals

Adult male Sprague-Dawley rats (250–300 g) obtained from the institute animal facility, were used for the present study. Animals were maintained under standard conditions of 12 h light/dark cycles, 22±1°C temperature and 60±5% humidity. Food and water were provided *ad libitum*. The experimental protocol was approved by the Institutional Animal Ethics Committee (IAEC) that is appointed and authorized by the Committee for the Purpose of Control and Supervision of Experimentation in Animals (CPCSEA) of the Division of Animal Welfare, under the Ministry of Environment and Forests, Govt. of India. A total of 83 rats were used in the study.

### Experimental paradigm

Animals were intracranially infused with rotenone to create hemiparkinsonian rat models. Only those animals showing more than 3 rotations per min following amphetamine administration (2.5 mg/kg, i.p.) on the 28th day post-infusion of rotenone were selected for the study. The animals were divided into 3 groups; control group with an equal volume of Hank's balanced salt solution (HBSS) infused into the striatum (n = 20), embryonic stem cells transplanted (ES) group (n = 24), and murine ES (mES) differentiated for 7 days transplanted (7 d) group (n = 30). Nine animals were used for testing recovery in 10 days differentiated cells (10 d) transplantation study. Transplantation surgery was done on the 32nd day after rotenone infusion. The animals were then maintained for 2 weeks and analysed for amphetamine- or apomorphine-induced rotation studies, elevated body swing test, immunocytochemistry, histology, striatal dopamine levels, RT-PCR and immunoblot experiments.

### Rotenone infusion into the median forebrain bundle (MFB)

Rats were anesthetised with ketamine and xylazine at 80 mg/kg and 8 mg/kg body weight, respectively. They were placed in a flat skull position on a stereotaxic frame (Stoelting, Wooddale, USA) with incisor bar fixed at 3.5 mm below the inter-aural line. Rotenone (10 µg in 2 µl) dissolved in DMSO and propane-1,2-diol in the ratio of 1∶1, was infused into the right MFB at a flow rate of 0.2 µl/min by employing a microinfusion pump consisting of a Worker Bee and Syringe pump (BAS, West Lafayette, USA). After stopping the pump, the probe was kept in the same position for 5 min for complete diffusion of the drug, and then slowly retracted. Stereotaxic coordinates of antero-posterior = −0.20 cm; lateral = 0.18 cm; and dorso-ventral = −0.82 cm from the Bregma point were followed for reaching MFB in rats [14].

### Intra-striatal transplantation of cells

Cells were trypsinized and counted to make aliquots of 5×10^5^ cells in 2 µl of HBSS (1×). These cells were then transplanted into the striatum of the lesioned side with rat stereotaxic coordinates, antero-posterior = +0.02 cm; lateral = 0.26 cm; and dorso-ventral = −0.50 cm from the Bregma point [14]. This was performed on 32nd day post-lesioning of the animals.

### Amphetamine-induced rotations

Rats were placed in Perspex, transparent cages (45 cm diameter and 45 cm height) after injecting amphetamine (2.5 mg/kg, i.p.) ipsilateral rotations were recorded for a period of 2 h [15]. These rotation evaluations were made once on the 28th day post lesioning to screen the animals, and again on the 44th day to compare the bias in rotations, in the lesioned and transplanted animals.

### Apomorphine-induced rotations

Animals were injected with apomorphine (0.5 mg/kg, s.c.) and the resulting rotations were counted for a period of 1 h. These rotations were performed on the 46th day post-lesion in the lesioned as well as in the transplanted animals.

### Elevated body swing test

Animals were held 2 cm from the base of their tail and elevated to 4 cm above the surface of the table. Animals were held at 90° to the surface of the table and this position was defined as no deviation, deviation of about 10° or less to either side was still considered as no deviation. A swing was recorded when the animals moved their head away from the vertical axis (angle >10°). Swings were recorded for 45 s [16]. After each swing is completed, the animals were made to return to the vertical position by letting their forelimbs touch the surface of the table. Results are expressed as the ratio of total number of contralateral swings∶ipsilateral swings recorded for 45 s. This experiment was performed on the 47th day post rotenone infusion.

### Semiquantitative PCR analysis

Total RNA was isolated from cells using TRI reagent (Sigma-Aldrich) following the protocol supplied by the manufacturer. An aliquot of 5 µg of total RNA was reverse transcribed using Mulv reverse transcriptase enzyme. The cDNA formed was diluted 10 times, and for PCR amplification 25 ng of the cDNA was used. PCR conditions included an initial denaturation at 94°C for 5 min, followed by 30 cycles of amplification reaction with denaturation at 94°C, annealing at 60°C and extension at 72°C for 20 s each. This was followed by a final extension at 72°C for 10 min. Equal amount of the product was electrophoresed on ethidium bromide containing 1.5% agarose gel. Bands were visualised in a ChemiDoc XRS gel documentation system (Bio-Rad) and densitometry was carried out using the software, Bio-Rad version 4.6.0. A list of the primers used in the study is provided in Table S1.

### Immunoblot

Protein was extracted from the samples and estimated as described by Lowry et al. [17], using BSA as the standard. An aliquot of 50 µg of protein was electrophoresed on a 10% SDS acrylamide gel and transferred to PVDF membrane. The membrane was then blocked with 10% (w/v) skimmed milk for 1 h, and incubated overnight with primary antibody at 4°C. The blots were then incubated with anti-rabbit horseradish peroxidase conjugated secondary antibody for 2 h, and developed with 3,3′-diaminobenzidine (DAB) and H_2_O_2_, and densitometry was performed using ImageJ software.

### Immuno-labelling of cells

Cells were grown and differentiated on gelatin coated coverslips. At the desired time point, the cells were fixed with 4% paraformaldehyde. These were washed with PBS and permeabilized with 0.1% Triton-X 100 for 5 min, washed with PBS again and blocked with 4% bovine serum albumin for 30 min. This was followed by overnight incubation with primary antibody at 4°C. The cells were extensively washed with PBS and incubated with fluorescence tagged secondary antibody for 1 h in the dark. The coverslips containing the cells were mounted on slides using an antifade mounting medium (UltraCruz™, sc-359850/Prolong gold antifade, Invitrogen), containing DAPI that stains nuclei, and were observed under an epi-fluorescence inverted microscope (Axiovert 200, Carl Zeiss). A detailed list of the antibodies used is provided as Table S2.

### Immunohistochemistry

Animals were transcardially perfused with 4% paraformaldehyde on the 48th day post lesioning of the brain region with rotenone. The brain was dissected out and kept in the fixative overnight and then transferred to 30% sucrose. Soon after the brain sank in the sucrose solution, it was taken out for cryosectioning. Cross sections of the brain were rinsed in cold PBS (0.1 M and pH 7.4) three times for 5 min each. These were then permeabilized with 0.4% Triton-X 100 for 30 min, blocked with 8% BSA and 0.1% Triton-X 100, and incubated overnight with primary antibody at 4°C. After washing, fluorochrome- or HRP- conjugated secondary antibody was added and incubated for 1 h in the dark. Following PBS wash, these sections were then mounted using antifade mounting medium for flurochrome secondary and were photographed. The HRP-tagged antibody treated sections were developed with DAB and H_2_O_2_, and were photographed.

### Dopamine measurement

Cells were washed thoroughly in PBS and sonicated in 50 µl of 0.4 M HClO4 containing 0.01% EDTA. The homogenates were kept on ice for 30 min for the proteins to precipitate, centrifuged at 18,000×g for 10 min at 4°C, and 10 µl of the supernatant was injected into a high performance liquid chromatography connected to an electrochemical detection system. The samples were run at 20 nA range of the detection sensitivity. The flow rate was 0.7 ml/min and the electrochemical detection was performed at 0.74 V [18]. Animals lesioned with rotenone and rotenone lesioned animals transplanted with mES or with differentiated mES were sacrificed on the 48th day post-lesioning and each striatum was dissected out and collected in separate labelled tubes. The tissues were sonicated in ice-cold HClO_4_ (0.1 M) containing 0.01% EDTA and kept on ice for 30 min, centrifuged at 18,000×g for 10 min at 4°C and 10 µl of the supernatant was injected into the chromatographic system. The sensitivity of the system was set at 200 nA range. The flow rate and electrochemical detection parameters were the same as was used for the cells.

### Cresyl violet staining

Slides were rehydrated in decreasing concentration of alcohol (absolute; 90%; 70%; 50%) for 1 min each, followed by 15 s dip in distilled water. Then they were stained with cresyl violet for 4 min and washed in distilled water for 15 s and followed dehydration in upgradation of alcohol (50% to absolute) for 30 s to 1 min. The slides were then cleared in xylene and mounted with DPX mountant.

### Cell counting

Phase-contrast images of the mounted slides were captured using the epi-fluorescence inverted microscope (Axiovert 200, Carl Zeiss). Cells in the ipsilateral and contralateral substantia nigra were counted using ImageJ software (For reference see, http://www.unige.ch/medecine/bioimaging/tricks/imagejtutorials/CellCounting.pdf).

### Statistical measures

The rotational and body swing bias ratio data were statistically evaluated for significance employing one-way ANOVA followed by Newman Keuls post-hoc analysis. Densitometry and neurochemical data were analyzed for significance using Student's t-test. Results are given as mean ± S.E.M. Value of *p*≤0.05 was considered significant.

## Results

### Stem cell markers are reduced following 7–10 days culture of mES in serum free media

**Figure 1:**
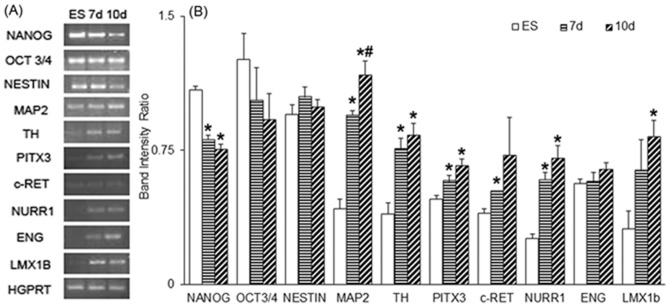
Changes in mRNA expression levels in cells following differentiation. Undifferentiated and differentiated embryonic stem cells (ES), and 7 days and 10 days differentiated (7 d; 10 d) cells were analysed for the mRNA expression of stem cells (NANOG; OCT3/4), neuronal (NESTIN; MAP2) and dopaminergic markers (TH; Pitx3; c-RET; NURR1; LMX1B; ENG). (A) Equal amount of cDNA was amplified for each gene and representative image of agarose gel bands obtained following electrophoresis. (B) Band intensities as normalised by hypoxanthine-guanine phosphoribosyl transferase (HGPRT; as housekeeping gene), showing relative gene expression. Results are Mean ± S.E.M, n = 3–4. *p≤0.05 vs. ES group, #*p*≤0.05 vs. 7 d group; Student's t-test.

**Figure 2:**
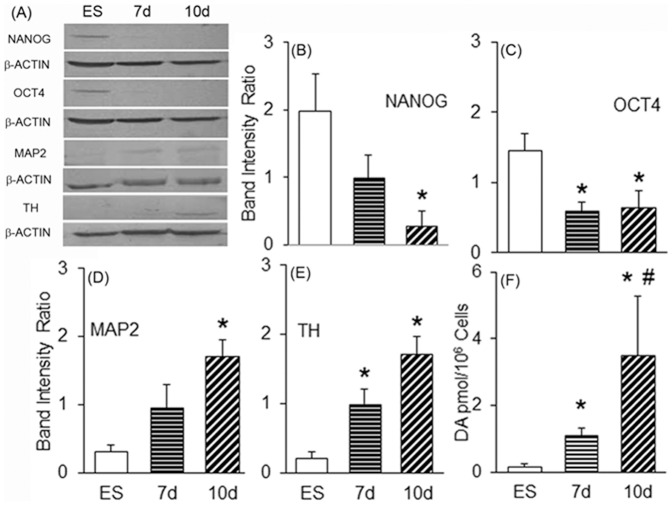
Characterization of differentiated cells. (A) Representative blots of cell lysates prepared from ES, 7 d and 10 d cells. A total of 50 µg of protein each were loaded into each well and transferred onto membrane and immunoblotted for β-actin (as loading control), NANOG, OCT3/4, MAP2 and TH. Bar graphs depicting the ratio of band intensities as normalised with β-actin for NANOG (B), OCT3/4 (C), MAP2 (D) and TH (E). (F) Dopamine (DA) content was assayed employing HPLC-electrochemistry in the homogenates prepared from ES, 7 d and 10 d cells. Results are Mean ± S.E.M, n = 3–4. *p≤0.05 vs. ES group, ^#^ *p*≤0.05 vs. 7 d group; Student's t-test. Data for DA was from 3–6 independent experiments. All the other abbreviations are as in Figure 1 legends.

**Figure 3:**
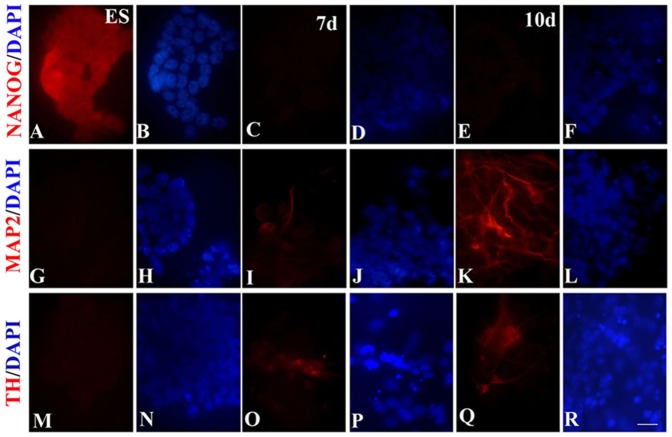
Immunocytochemical localization of stem cells, neuronal and dopaminergic markers. Immunostaining for pluripotency marker, NANOG for ES (A), 7 d (C) and 10 d (E), co-stained for nuclei with 4,6-diamidino-2-phenylindole (DAPI) (B,D,F). Microtubule associated protein (MAP2) is used for showing the presence of neurons in ES (G), 7 d (I) and 10 d (K), co-stained with DAPI (H,J,L). To see if dopaminergic neurons are present, the ES (M), 7 d (O) and 10 d (Q) cells were stained for tyrosine hydroxylase (TH), a rate limiting enzyme for dopamine synthesis. DAPI was used for staining nuclei (N,P,R). Nuclear stain, DAPI showed the total number of cells for each field. Scale bar 20 µm. All the other abbreviations are as in Figure 1 legends.

mES were differentiated by keeping in serum and LIF free media and at an optimum plating density [13], for 7 to 10 days. The 7 days differentiated cells showed small rounded colonies with very few neurites outgrowth, while the 10 days differentiated cells showed extensive neurites, compared to the ES. The mRNA expression pattern (Figure 1A) of stem cell marker NANOG was found to be significantly reduced, whereas OCT3/4 was unaffected during 7–10 days of default neurogenesis in serum free media (Figure 1A, B). The protein levels of NANOG and OCT3/4 were found to be inhibited on days 7 and 10 (Figure 2A). Statistical significance on the ratios of the band intensities of OCT3/4 to that of β-actin was found to be significant for both the days, but only on 10 days for NANOG in the immunoblot analysis (Figure 2B,C). Immunocytochemical analysis showed similar reduction in immunostaining of NANOG (Figure 3A,C,E) in most of the cells in culture demarcated by DAPI staining (Figure 3B,D,F) on 7 and 10 days.

### Neuronal markers were up-regulated following 7–10 days culture of mES in serum free media

mRNA expression of one of the neuronal markers studied, the microtubule associated protein (MAP2), but not the initiating neuronal differentiation marker, NESTIN was found to be upregulated on days 7 and 10 in the default differentiating media (Figure 1A,B). MAP2 protein level was also found to be increasing with time in the cells grown in serum free media (Figure 2A) and was significantly elevated by day 10 (Figure 2D), which was also discernible in immunocytochemical studies conducted on these days (Figure 3G,I,K). The percentage of MAP2 positive cells after 7 days differentiation was 21.9±2.1, while after 10 days it was 41±5.3 (Figure S1).

### Dopaminergic neuronal markers were up-regulated in murine ES by default neurogenesis

NURR1, a key molecule in the maintenance of the dopaminergic system of the midbrain; LMX1b and PITX3, a LIM homeodomain and a paired like homeodomain transcription factor/regulator respectively known to have dopaminergic lineage; c-RET, a proto-oncogene associated with mid-brain dopaminergic neuronal development; the rate limiting enzyme of dopamine synthesis, TH; and engrailed-1 (ENG) known to have a role in spatial development of the midbrain, were analyzed in mES, 7 d and 10 d cells. While mRNA expression of ENG was unaffected, all the other genes were over-expressed either on 7days or both the days of analysis (Figure 1A). By taking hypoxanthine-guanine phosphoribosyl transferase (HGPRT) expression levels as house-keeping gene, it has been found that TH, PITX3, and NURR1 were highly expressed in cells from both these days, whereas c-RET and LMX1b were upregulated only on 7th and 10th day respectively, and ENG was unaffected (Figure 1B). Immunoblot revealed high levels of TH protein (Figure 2A), which was statistically significant for both the days when adjusted to β-actin protein levels (Figure 2E). TH expression was also seen to be higher in neurons of 7 and 10 days (Figure 3M,O,Q). The percentage of TH positive cells was higher in both the groups but not significantly different from each other with 10.5±2.8% in 7 d cells and 17.5±4.2% in 10 d cells (Figure S1).

**Figure 4:**
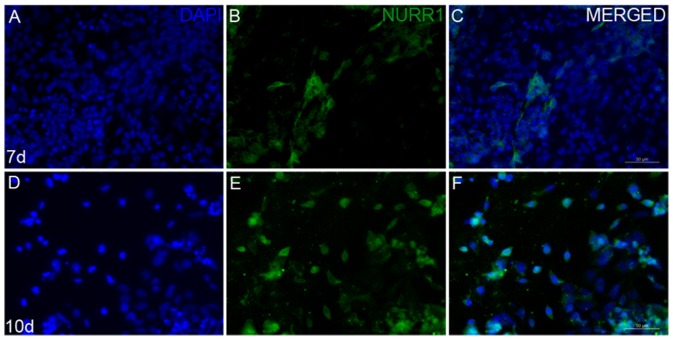
Expression of NURR1 in 7 and 10 days differentiated cells. Immunocytochemistry showing the levels of NURR1 proteins as a midbrain dopaminergic neuronal marker in 7days (A–C) and 10 days (D–F) differentiated cells. DAPI stained the nuclei (A,D). Scale bar is 50 µm.

**Figure 5:**
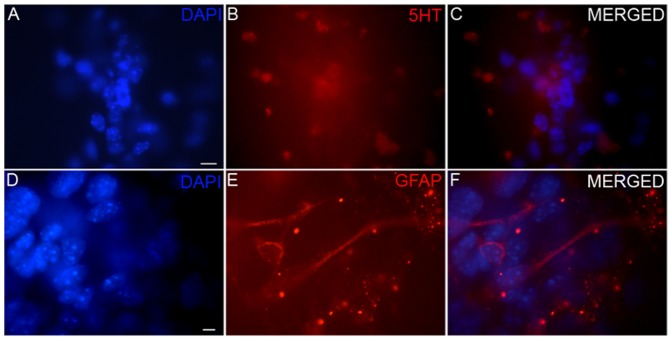
Presence of serotonin and GFAP positive cells in the differentiated cells. Immunocytochemical localization of serotonin (5-HT; B,C) and glial fibrillary acidic protein (GFAP; E,F) in 7 days differentiated cells showing glial (E) and few serotoninergic neurons (B) in the culture. Scale bar 10 µm.

Expression of NURR1 protein was seen to increase with the increase in days of differentiation (Figure 4B,E). This was evident from the nuclear staining with DAPI (Figure 4A,D), which showed more number of cells but less number of neurons stained for NURR1 proteins (Figure 4C,F). A study for the presence of other neuronal or glial cell markers in the differentiated cells revealed, few 5-HT (Figure 5B,C) and GFAP positive cells in the 10 d cells (Figure 5E,F), while 7 d cells did not show any reactivity for this neurotransmitter amine (data not shown).

### Difference in behavioural parameters of experimental groups

It has been recently demonstrated that neuronal survivability and viability is directly related to a great extent on neuronal maturity, and relatively maturing neurons will have higher survivability in an environment in the host brain that is rich in excitatory amino acids and other debilitating factors resulting from oxidative stress and immunological responses to tissue trauma [19]. Since 10-days differentiated cultures showed a lower population of progenitors and a higher population of mature neurons as compared to 7-days differentiated cells (Figure S1), the latter was considered for transplantation studies. The 10 d cells had more of neurons as seen by the count of MAP2 positive cells, and more of TH positive cells (Figure S1). For control we had mES transplanted animals.

**Figure 6:**
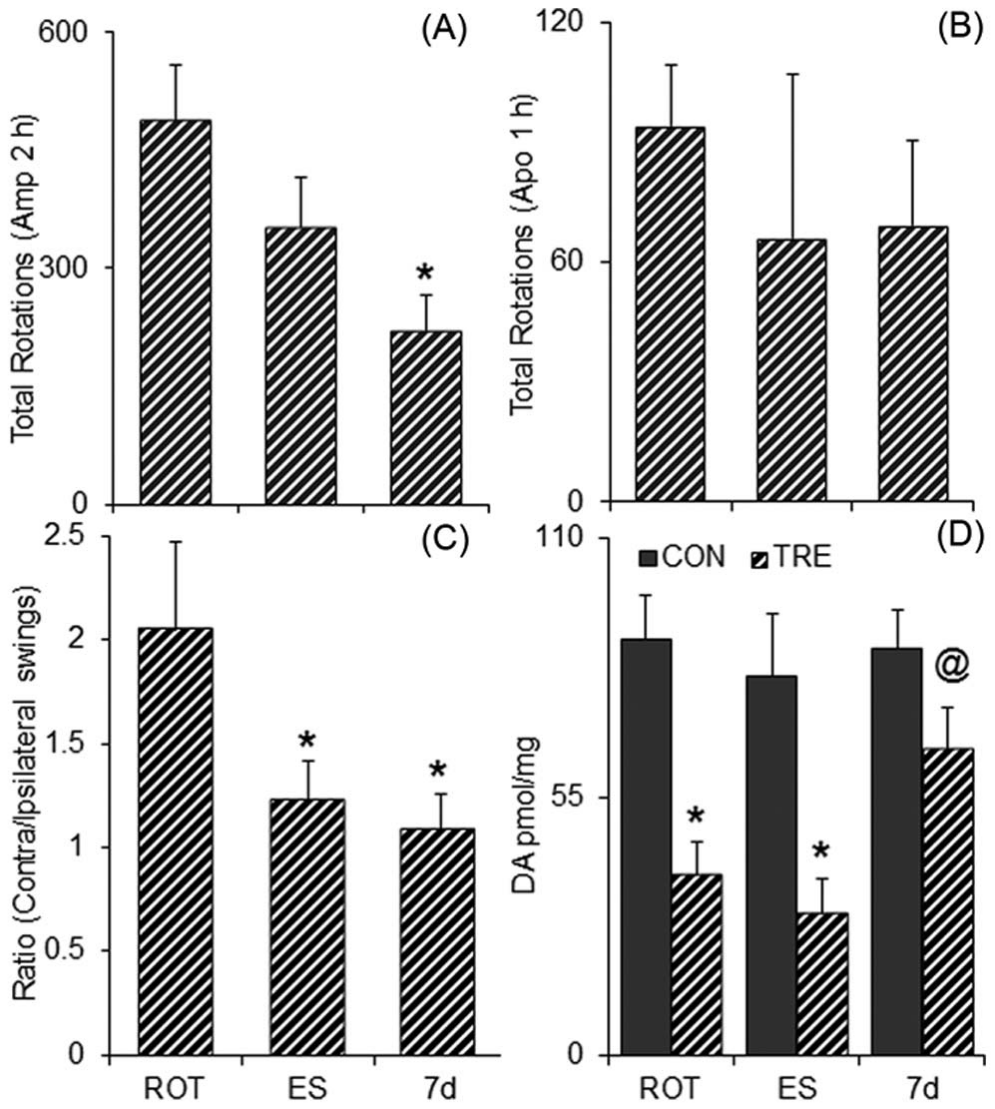
Transplantation-induced functional recovery. Bar graphs in (A) and (B) depict the number of unilateral rotations in hemiparkinsonian rats infused with vehicle only without any cells (ROT), or transplanted with 5×10^5^ embryonic stem cells, undifferentiated (ES) or 7 days differentiated cells (7 d) in the striatum, and treated with amphetamine (Amp) or apomorphine (Apo) respectively on 14th day or 16th days post-transplantation. Amphetamine caused ipsilateral rotations, whereas apomorphine caused contralateral rotations. (C) Preferential bias in body swings, recorded on the 47th day post-lesion or 17th day post transplantation. *p≤0.05 as compared to the pre-transplanted values of rotations, which was similar to vehicle infused group, n = 6–7. (D) Striatal DA levels in the ROT, ES and 7 d groups. *p≤0.05 as compared to the control contralateral side, ^@^ *p*≤0.05 as compared to ES transplanted striatum, n = 5–6.

Systemic administration of amphetamine on the 14th day post transplantation caused ipsilateral rotations (∼250 rotations/h) in rats (Figure 6A) that have been unilaterally lesioned by rotenone infusion into MFB, accompanied by severe depletion of ipsilateral striatal DA levels (Figure 6D). While the animals transplanted with mES exhibited insignificant reductions in the rotations following amphetamine, the rats that received 7 d cells as transplant in the ipsilateral striatum, showed significantly fewer number of rotations (about 110 rotations/h) (Figure 6A). On the 16th day the animals were analysed for apomorphine-induced rotations. Rats unilaterally lesioned with rotenone showed contralateral bias in rotations, which amounted to about 120 rotations/h (Figure 6B). Neither the mES, nor the 7 d cell transplanted rats exhibited any reductions in the contralateral bias in rotations (Figure 6B). To check if the behavioural outcome is reflected in a non-pharmacological test we performed the elevated body swing test. Rotenone-lesioned control animals showed contralateral bias in swings. The ratio of contralateral∶ipsilateral swings was reduced in both ES and 7 d transplanted group (Figure 6C).

### Intra-striatal transplantation of 7 d cells led to elevation of striatal dopamine levels

The animals were sacrificed on the 18th day post transplantation. HPLC analysis of the ES implanted striatum revealed no change in DA levels as compared to the striatum lesioned with rotenone (Figure 6D), whereas a significant increase in the levels of DA was found in 7 days differentiated cell transplanted animals as compared to rotenone infused control or ES transplanted animals (Figure 6D). However, 10 d cells were transplanted to reconfirm earlier reports and we also found that they were not capable of reducing the amphetamine induced rotations and bring back the striatal DA levels (Figure S3).

### TH immunohistochemistry and cresyl violet staining of the graft

**Figure 7:**
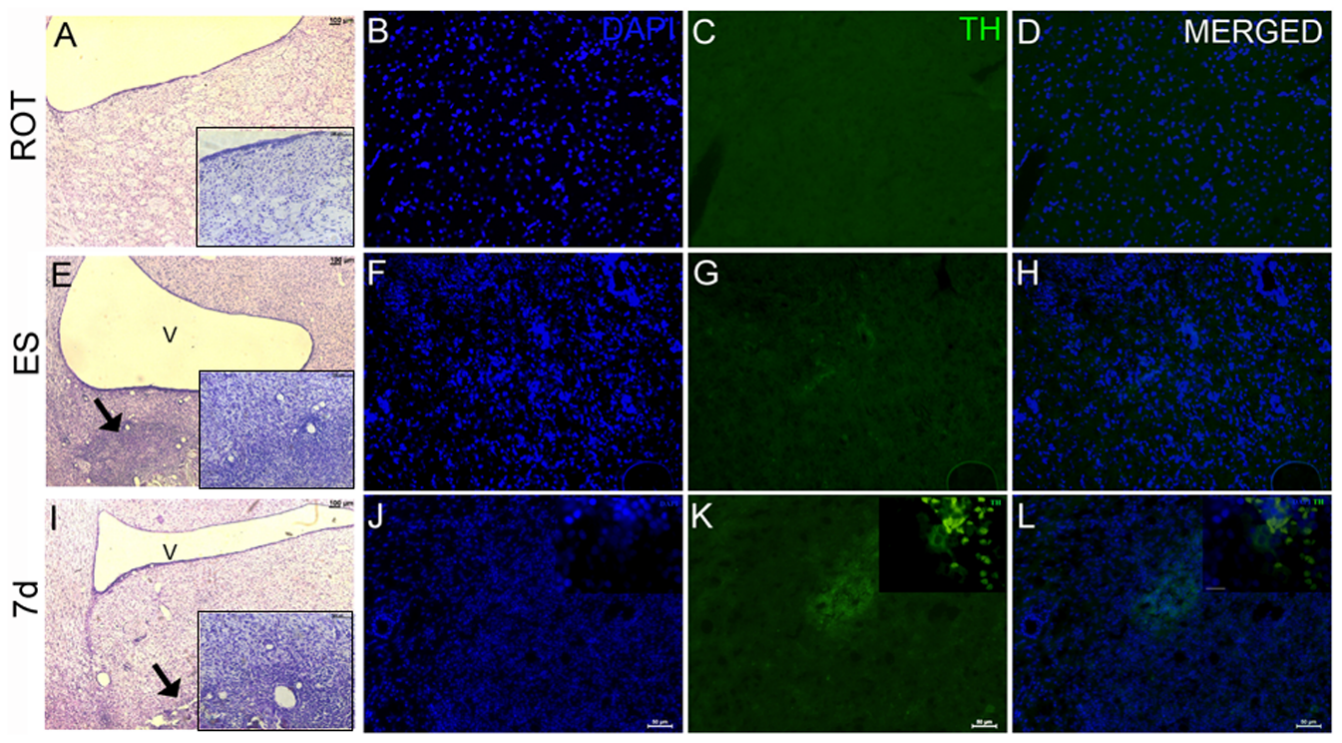
Characterization of the grafted cells in the striatum. Striatal sections of the rotenone-infused control (ROT), embryonic stem cells transplanted animal (ES) and 7 days differentiated cells transplanted animal (7 d) were processed for presence of dopaminergic cells positive for tyrosine hydroxylase (TH) immunoreactivity. Sections were stained with cresyl violet (A, E and I) for localization of neurons (Scale bars for both inset as well as the main frame are 100 µm), and with DAPI for nuclei (B,F,J). Sham transplanted (ROT; B–D); undifferentiated embryonic stem cells transplanted (ES; F–H) and 7 days differentiated cells grafted (7 d; J–L) striatal images are shown. Scale bar is 50 µm for these figures. Scale bar for the magnified images showing TH positive cells, in the right hand corners of the lower panel is 10 µm.

Cresyl violet staining showed clear neuronal distribution in the striatum of rotenone infused, sham transplanted animals (Figure 7A). In the ES transplanted and the 7 d differentiated cells transplanted group, dense cresyl violet staining was seen in the graft core (Fig. 7E,I). The 7 d cells' graft in the striatum showed few TH positive neurons (Figure 7J–L), which were not seen in the vehicle-infused control (Figure 7B–D) or in the ES grafted striatum (Figure 7F–H). The TH immunoreactive cells were restricted to a small part of the graft, and showed no visible interaction (inset-lower panel) with the host striatum by the day the analysis was made (Figure 7K,L).

### Astrocytosis in the periphery of the murine cell graft

**Figure 8:**
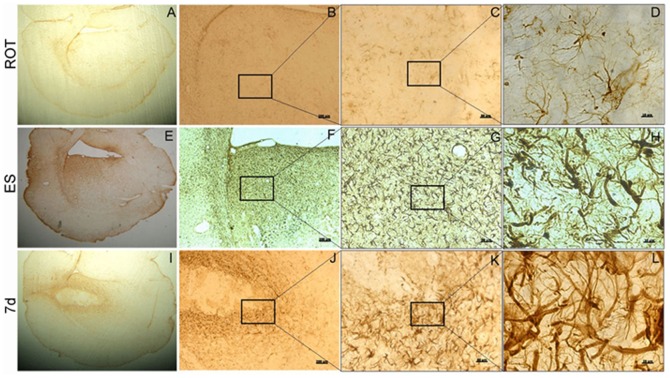
Astrocytosis in differentiated cells implanted striatum. Striata, ipsilateral to the side of rotenone infusion were grafted with vehicle without any cells (ROT; A–D), embryonic stem cells undifferentiated (ES; E–H) or 7 days differentiated cells (7 d; I–L), were stained for the astrocyte marker glial fibrillary acidic protein (GFAP) immunoreactivity. Increased immunostaining for GFAP is seen only in the ES and 7 d cells grafted striata. A,E and I are low magnification images, whereas D,H,L and C,G,K are magnified images from the ‘boxes’ each from the previous figures, as marked in the same lane. A,E,I are 5× magnification, whereas scale bars in B,F, and J are 200 µm; in C,G, and K are 50 µm, and D,H, and L are 10 µm.

**Figure 9:**
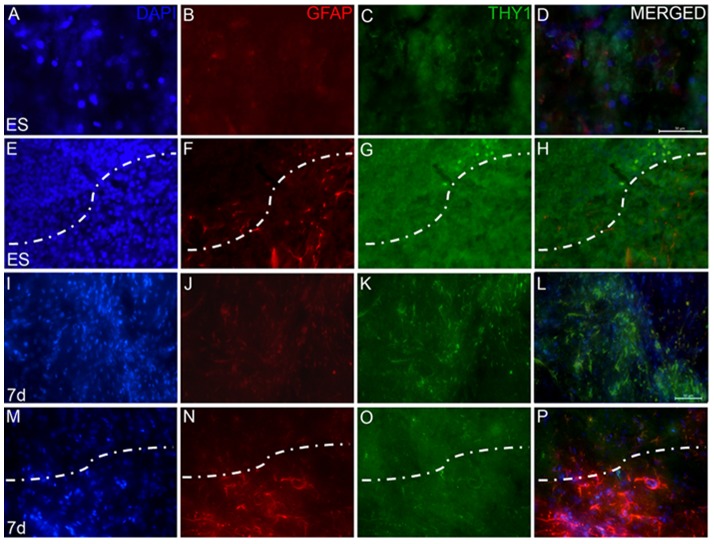
Co-localization of GFAP and murine specific thymocyte antigen 1. Striatal sections of 20 µm thickness of embryonic stem cells, undifferentiated (ES; A–H) and 7 days differentiated cells (7 d; I–P) transplanted group were co-stained for glial fibrillary acidic protein (GFAP; B,F,J,N) and murine specific thymocyte antigen 1 (THY1; C,G,K,O) to examine the origin of these glial cells. Figures A–D and I–L are images from locations within the graft, whereas E–H and M–P are locations along the margin of the graft, showing the host tissue and graft. The dotted lines demarcate the margin. GFAP staining is seen outside the graft margin, whereas THY1 positive cells are seen only inside the graft. Scale bar 50 µm.

Immunohistochemical analyses for an astrocyte marker, glial fibrillary acidic protein (GFAP) in the ipsilateral striatum of the rotenone infused animal that received only the vehicle, showed a number of cells stained for this marker (Figure 8A–D). The staining was definite, sharp, and the cell body and the fine fibres were stained positively (Figure 8D). mES implanted region of the striatum revealed robust changes in the fibres, which were found to be stouter as compared to normal striata, and took up intense staining for GFAP (Figure 8E–H). Seven days differentiated neuronal graft in the striatum exhibited similar morphology of the astrocytes as ES transplanted group (Figure 8I–L). Striatal sections passing through the graft were co-immunolabelled with GFAP and murine thymocyte antigen 1 (THY1), so as to examine if the murine antigen co-localizes with glia. GFAP antigen was found to be located outside or at the margin of the graft (Figure 9F,H,N,P), whereas THY-1 antigen was found only within the graft (Figure 9G,H,O,P).

### Accumulation of microglia in the grafted striatum

**Figure 10:**
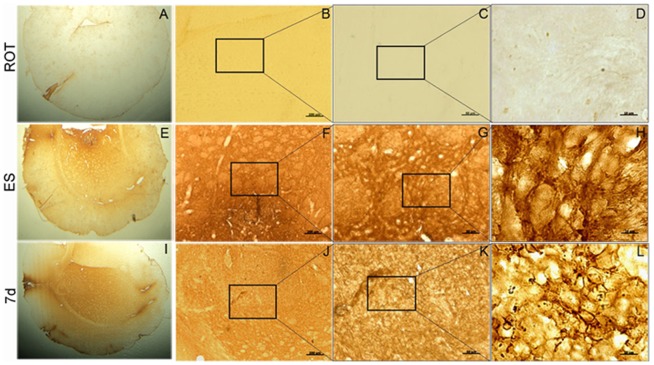
Microglial immunorectivity in striatal grafts. Ipsilateral striatum of sham (A–D), ES (E–H) or 7 days differentiated cells (I–L), transplanted groups were stained for CD11b immunoreactivity. No immune-staining for CD11b was detectable in vehicle, without any cells infused striatum (see A–D). Increased immunostaining for the microglial marker, CD11b is seen only in the ES (E–H) and 7 d cells (I–L) grafted striata. Each figure in CGK and D,H,L is the magnified versions of the marked regions of the figure from the previous figure in the same lane. Magnifications are as stated in Figure 8 legends.

Immunoreactivity for a marker for microglia, CD11b in the striatum ipsilateral to rotenone infused side revealed no staining (Figure 10A–D), whereas in the ES grafted striatum, clear indications of gliosis was discernible (Figure 10E–H). Stronger gliosis as revealed by more number of microglia stained for CD11b was visible in the striatal region where 7 d cells were transplanted (Figure 10I–L).

**Figure 11:**
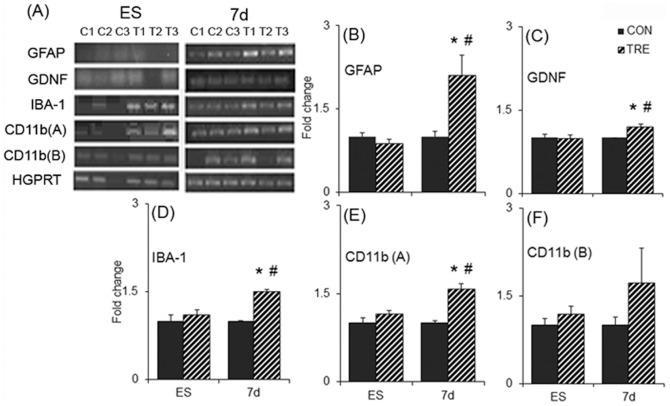
mRNA expression of certain glial markers in the striatum. (A) Gels showing amplified cDNA bands for different primers. The cDNA was prepared from the mRNA isolated from the control and treated sides of the striatum of the embryonic stem cells, undifferentiated (ES) and 7 days differentiated (7 d) cell transplanted groups. Ratios of the band intensities for GFAP (B), GDNF (C), IBA-1 (D), CD11b-A (E) and CD11b-B (F) as normalised with hypoxanthine-guanine phosphoribosyl transferase (HGPRT) and fold change of band intensities of the treated side compared to the control side was calculated for each animal. Results are presented as Mean ± SEM. *p≤0.05 vs control side of the same group, ^#^ *p*≤0.05 vs treated side of the ES transplanted group, n = 3.

Semi-quantitative PCR carried out for the mRNA levels of ionized calcium-binding adaptor molecule 1 (IBA-1), and two variants of cluster of differentiation molecule 11b [CD11b(A) and CD11b(B)], three of the markers of reactive microglia showed significant increase in its expression pattern in the 7 d cells transplanted striatum as compared to mES transplanted group (Figure 11). The ratios of band intensities of GFAP, IBA-1 and CD11b(A) (Figure 11B,D–E) with that of the level of expression of HGPRT was found to be significantly higher in 7 d transplanted striata as compared to mES implanted striata. However CD11b (B) expression showed no significant change in any of the groups (Figure 11F).

### GDNF in the grafted striatum and SN ipsilateral to the transplant

**Figure 12:**
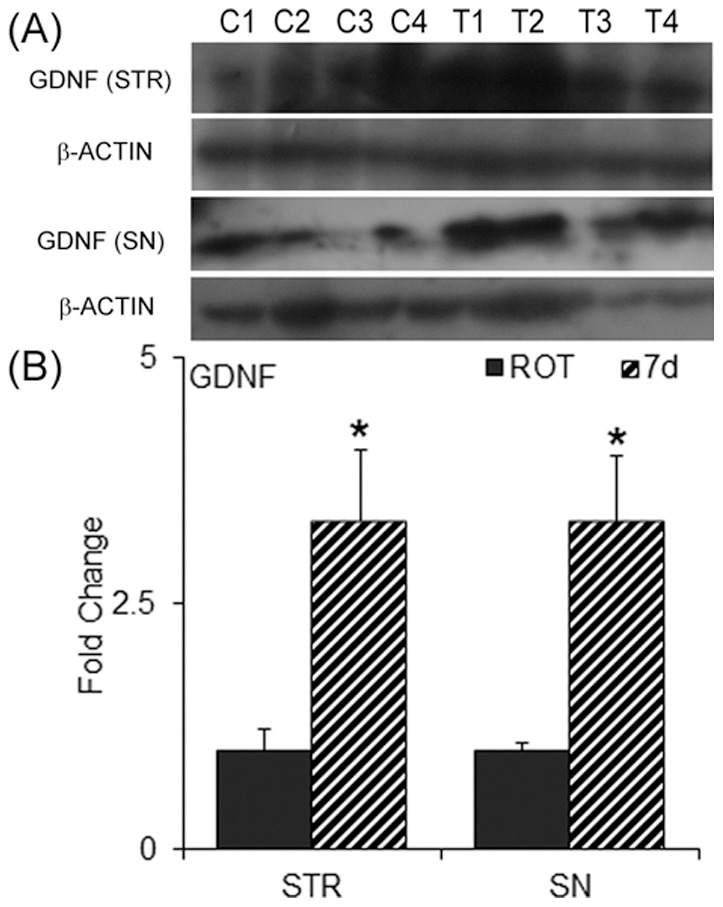
Neurons in the nigra of the animals that received unilaterally grafts in the striatum. Sections passing through the substantia nigra pars compacta were cut and immunostained for tyrosine hydroxylase (arrows; ipsilateral nigra) (A–C) or stained for neurons with cresyl violet (D–F). The sections passing through nigral region of the midbrain of rats that received the vehicle, without cells (A, D), undifferentiated embryonic stem cells (ES; B,E) or 7 days differentiated ES (7 d; C,F). There appeared a significant percentage of improvement in the total number of neurons in the ipsilateral substantia nigra relative to the contralateral side (G), and in TH-positive dopaminergic neurons (H). Results are presented as Mean ± SEM. *p≤0.05, as compared to control or ES transplanted group. Sections from 3 different brain samples were considered for each group.

mRNA expression of GDNF was found to be significantly higher in 7 d transplanted striata as compared to mES implanted striata (Figure 11A,C), along with a concomitant increase in the protein level (Fig. 12A,B). We also found an increase in the protein levels of GDNF in the SN ipsilateral to the side of the 7 d transplanted group, when compared to the rotenone lesioned ipsilateral SN (Figure 12A,B). The increase was found to be almost 3-fold.

### Protection of nigral dopaminergic neurons

**Figure 13:**
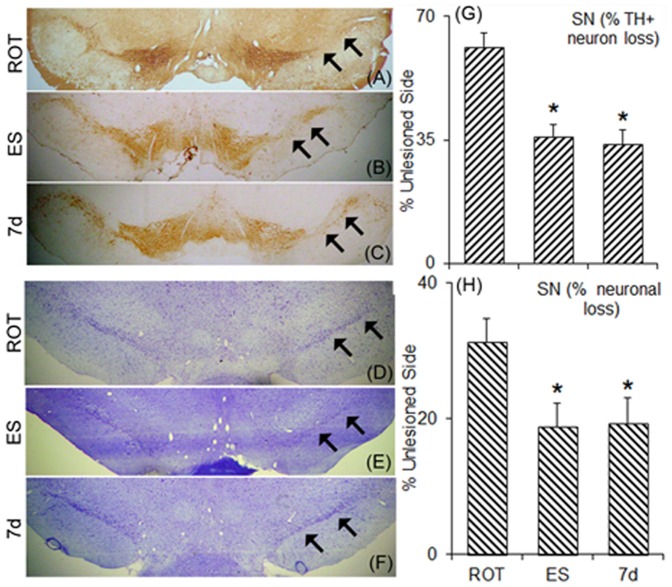
Protein levels of GDNF in the striatum and in the substantia nigra (SN). The ipsilateral striata and SN were dissected out sham grafts (ROT) and 7 days differentiated cells transplanted animals, and immunoblot images from four different animals are provided (A). A ratiometric analysis of the GDNF band intensity to β-actin, followed by fold changes with respect to rotenone is plotted (B). There was approximately 3-fold increase in both the regions, striata and SN as compared to rotenone treated control. Results are represented as Mean ± SEM, *p≤0.05 as compared to the control group, n = 4–6.

TH immunopositive neurons in the ipsilateral and contralateral SNpc were counted and determined the percentage recovery in the number of neurons following ES or 7 days differentiated cells transplantation. It has been found that rotenone infusion into the median forebrain bundle caused a significant loss (about 65%) of TH expression in nigral cells (Figure 13A) with only a 33% cresyl violet loss of neurons in nigra. Intrastriatally mES or 7 days differentiated cells transplanted animals showed significant recovery in the number of neurons in the ipsilateral SNpc as stained for cresyl violet (Figure 13F), and in TH positive cells (Figure 13C). The treatment prevented loss of about 30% of TH expression, and protected about 10% of the nissl stained cells.

Presence of proliferating cell nuclear antigen (PCNA) immunoreactivity was clearly discernible both in the ES as well as in the 7 days differentiated cells grafted striatum (Figure S2).

## Discussion

The mechanism of differentiation of ES into neurons in a serum free condition is thought to be a default process, in which the absence of any cue for development of epidermis, endoderm or mesoderm, results in neuralization of the ES [20]. Based on this cue, Lenka and Ramasamy [21] have reported successful differentiation of mES into neurons in serum free condition. The usage of such a protocol minimized the variation factor usually associated with the use of animal products for culture [22], thereby avoiding cross species contamination [12] making the cells more suitable for transplantation. There are reports suggesting the differentiation of ES grown as adherent monolayers in serum free condition involves synthesis of retinoic acid from the vitamin A present in the medium [23]. With the inhibition of this retinoid signal, cells differentiate towards mesodermal fate through activation of Nodal, which in turn activates Wnt signalling, resulting in ES differentiation towards a non-neural fate. The default mechanism has been shown to generate neural progenitor cells which are capable of differentiation *in vitro* and of transplantation [24], where the myelination potential of these cells was assayed. Our goal was to assess the efficacy of this procedure in generating DA neurons and the post-transplantation potential for benefits in an experimental PD model.

OCT3/4 and NANOG are key regulators essential for the formation and/or maintenance of the inner cell mass during mouse pre-implantation development and for self-renewal of pluripotent ES [25], [26]. Therefore a decrease in their expression as found in the present study denotes differentiation of ES. Neuronal nature of the differentiated cells was confirmed by the increase in the levels of mRNA levels of neuronal markers such as MAP2 and NESTIN, as well as increase in MAP2 immunoreactive cells. MAP2 is involved in microtubule assembly, which is an essential step in neurogenesis. It serves to stabilize microtubules growth by cross-linking them with intermediate filaments [27], thereby forming mature neurons. Increased expression of LMX1b and PITX3 (bicoid-related homeodomain-containing transcription factors) are involved in establishing the DA phenotype. Presence of TH and NURR1 positive cells in our culture confirms that some of the cells are dopaminergic in nature. It has been shown that human ES-derived neuronal progenitors could be differentiated into neurons, exhibiting midbrain DA phenotype, expressing Aldh1a, PTX3, NURR1 and LMX1a by repeated passaging in the presence of basic fibroblast growth factor and medium conditioned with PA6 stromal cells [28]. Studies have shown that DA neurons acquiring SNpc specificity requires PITX3 to be induced [29]. Presence of most of these transcription factors in the culture suggests the possibility of generation of midbrain DA neurons amongst the differentiated cells. Additionally the cells were also capable of synthesising DA as measured by HPLC-electrochemistry, confirming the functional competence of the cells.

Most of the experimental transplantation studies have been undertaken in 6-hydroxydopamine (6-OHDA)-induced [30], [31], [32] or 1-methyl-4-phenyl-1,2,3,6-tetrahydropyridine (MPTP)-induced [33], [34], [35] models of PD. Literature reveals only a couple of reports of transplantation studies in rotenone model of PD [36], [37], which is unique in terms of its progressive nature [38] and protein aggregation pathology [39]. We have chosen the site of infusion of the neurotoxin to be MFB, which induces ipsilateral or contralateral rotations when animals are challenged respectively with amphetamine or apomorphine, unlike intranigral or intrastriatal infusion of rotenone [40]. Therefore we were assured of selective loss of nigrostriatal pathway since amphetamine and apomorphine challenge respectively is an indicator of presynaptic DA release, and postsynaptic supersensitivity, and so true signs of destruction of nigrostriatal pathway [41].

The cells were transplanted ectopically in the striatum, the site of DA release, as it is well established that transplanting cells at their ontogenic site, SNpc, fails to extend axons up to the striatum thereby not showing desired results [42]. Enriched neurosphere cultures could relive in a hemiparkinsonian rat models [43] and so we thought it prudent to transplant a culture rich in progenitor population. The 7 d cells had relatively higher expression of neuronal progenitors with some mature neuronal cells, unlike 10 d cells which showed reduced expression of NESTIN and increased expression of MAP2. For control we used ES to see if the effect is specific to differentiated cells.

The animals that received the 7 d grafts showed improvement in amphetamine-induced rotations as compared to the rotenone infused, cell-less vehicle transplanted control. This suggested that DA-rgic cells surviving in the striatum are responsive to amphetamine administration. However apomorphine failed to show any beneficial effects of 7 d cell transplantation in the present study. This could be due to the subsistence of supersensitivity of the postsynaptic DA receptors, despite presence of DA producing cells within the striatum. This is clearly due to the short time period, only two weeks made available for overcoming the supersensitivity.

To examine the effect of transplantation on a drug free behavioural parameter, we did the elevated body swing test. Earlier reports from our laboratory have shown that unilateral intra-MFB rotenone infusion results in ipsilateral body swings, however in the present study we found increase in the contralateral swings [44]. We assume that this is because of a subtle difference in the experimental protocol; animal response time in the previous study was 5 seconds, whereas in the present study it was 45 seconds. The ratio of contralateral to ipsilateral swings was found to be >2 suggesting a bias towards the contralateral side. This bias could be reduced significantly in both the transplanted groups where the ratio was more close to 1.

Both the 7 d differentiated cells and ES transplanted striata exhibited an increased number of CD11b and GFAP positive cells surrounding the graft. Together with these, the mRNA expression levels of IBA-1, CD11b, GFAP and GDNF were found to be increased significantly in the 7 d cells transplanted striatum. Since our data show that GFAP positive cells are of host origin, we assume, the over-expression CD11b and IBA-1 positive cells elucidates host response to the foreign cells (murine origin) that have been grafted. This was expected since we have not used any immunosuppressive agents.

Microglia play critical role in neuroinflammation, host defence and probably neurodegeneration, essentially through the release of reactive oxygen species by activation of NADPH oxidases [45]. Activated microglia, positive for CD11b, are known to elicit graft rejection by secretion of cytokine IL-2, which stimulates T-cell proliferation, and TNF-α which recruit monocytes and lymphocytes [46]. This probably explains the low survival of cells in the graft, especially the TH positive cells in the transplanted striata. Additionally, proper understanding of graft-induced tissue damage and reactions of the brain immune system is essential for brain transplantation studies. This is since cell migration, survival, death, toxicity and immune responses differ with respect to the types of cells and environment of cells that are grafted [47].

mRNA levels of IBA-1, the resting microglial marker, and CD11b the reactive microglia marker, were significantly less in the ES transplanted group compared to 7 d transplanted group. This finding is explained by a study that reports; undifferentiated ES show stimulated release of NANOG which has been shown to down-regulate the mRNA and protein levels of pro-inflammatory cytokines IL-1β, TNF-α and IL-6, and reduce the transcriptional activity of NF-κB [48], thereby acting as an anti-inflammatory agent.

Another interesting aspect of our study is the significant protection of the neuronal perikarya of the ipsilateral SNpc dopaminergic neurons whose terminal region received mES undifferentiated or 7 days differentiated mES. We suspect this might be due to the increased GDNF, which is known to be retrogradely transported from the striatum into SNpc [49], since we found an increase in the protein levels of GDNF in the ipsilateral striatum and SN of the 7 d transplanted group compared to rotenone-infused control SN and striatum. Additionally, we also found an increase in the mRNA levels of GDNF in the striatum of both ES and 7 d transplanted groups. GDNF is known to help in the survival of midbrain dopaminergic neurons and has been used as a neuroprotective agent in the PD [42]. This neurotrophic factor is shown to cause stem cell survivability by reducing the expression of caspase 3, and enhancing the expressions of Bcl-2, NURR1 and PITX3 in the culture [50]. In the host brain these cells helped to derive TH-positive neurons, and improved behavioral and neurochemical deficiencies in 6-OHDA lesioned rats, via stimulation of GDNF receptor, GFRα1 [50]. Furthermore, GDNF has also been shown to help sprout neurites in the DA terminal region, and to increase its synthesis in human PD brain following its long-term continuous infusion into globus pallidus [51].

One of our major concerns is the presence of PCNA positive dividing cells in the graft which, in the long run, might lead to tumorous growth. Some approaches have been proposed to avert the problem, such as prolonged maturation of cells in culture [52], multiple passaging of cells in the differentiating media [53], use of specific inhibitors of the differentiating signal receptors of the parent cells [54], and selectively eliminating undifferentiated cells from the cultures [55]. Currently we are investigating multiple passages of ES in differentiating culture media to reduce undifferentiated cell population, and manually segregate the undifferentiated cells using magnetic sorting methods.

To conclude, the present study strongly suggests that ES grown in a serum free condition can differentiate into neural progenitors, and ultimately differentiate into DA-rgic cells *in vitro*, as well as within the graft, *in vivo*. Neuronal progenitor cells in these differentiated cells when transplanted into the striatum of hemiparkinsonian rats not only develop into mature neurons, restore the neurotransmitter levels, and produce protective glial cells. Further study should be designed to address the shortcomings such as contamination of unwanted cells in the grafted population, activation of microglia at the site of transplantation, and the low level of transplantation recovery observed.

## Supporting Information

Figure S1 **Number of neuronal and tyrosine hydroxylase (TH) positive cells in 7 days (7 d) and 10 days (10 d) differentiated stem cells.** MAP2 (neuronal marker) and TH (dopaminergic neuron marker) were immunostained, and were observed under a fluorescence microscope. The percentage of neurons was calculated against the total number of cells (DAPI count) in the field. Results are presented as Mean ± SEM. *p≤0.05, from 3 different experiments.
(TIF)

Figure S2 **Dividing cells in the grafts.** Ipasilateral striata have been processed for immune reactivity for proliferating nuclear antigen (PCNA) for the presence, if any of dividing cells. DAPI has been used to locate nuclei in the cells. Note that the middle row images that represent the undifferentiated embryonic stem cells transplanted striatum contain more PCNA positive cells than in the striatum that received 7 days differentiated cells (Lower panel). The top panel contains images from animals that were lesioned with rotenone (ROT) and received only the culture medium, without any cells. Magnification scales in figures are 10 µm.
(TIF)

Figure S3 **Mature neurons when transplanted do not show recovery.** Ten days differentiated embryonic stem cells (10 d) were transplanted into the ipsilateral striatum of unilaterally lesioned animals. No improvement was seen in amphetamine induced rotational behaviour in the transplanted animals as compared to the sham control (A) or in the levels of the striatal dopamine levels (B). Results are presented as Mean ± SEM. *p≤0.05, n = 3.
(TIF)

Table S1 **List of primers used for Semi-quantitative PCR.**
(DOC)

Table S2 **List of antibodies and their sources used for Immuno-cytochemistry and Immuno-blotting studies.**
(DOC)
