# Supplementary figures and images for: Engraftment of Mouse Embryonic Stem Cells Differentiated by Default Leads to Neuroprotection, Behaviour Revival and Astrogliosis in Parkinsonian Rats
Source: PLoS One. 2013 Sep 12;8(9):e72501. doi: 10.1371/journal.pone.0072501 (PMC3772067; doi:10.1371/journal.pone.0072501)

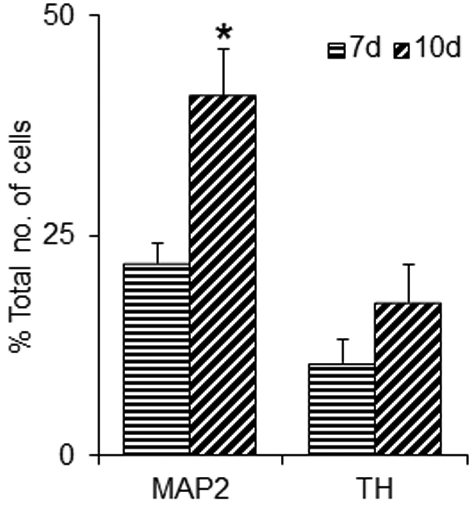

Supplement: Figure S1 — Number of neuronal and tyrosine hydroxylase (TH) positive cells in 7 days (7 d) and 10 days (10 d) differentiated stem cells. MAP2 (neuronal marker) and TH (dopaminergic neuron marker) were immunostained, and were observed under a fluorescence microscope. The percentage of neurons was calculated against the total number of cells (DAPI count) in the field. Results are presented as Mean ± SEM. *p≤0.05, from 3 different experiments. (TIF) [file pone.0072501.s001.tif]

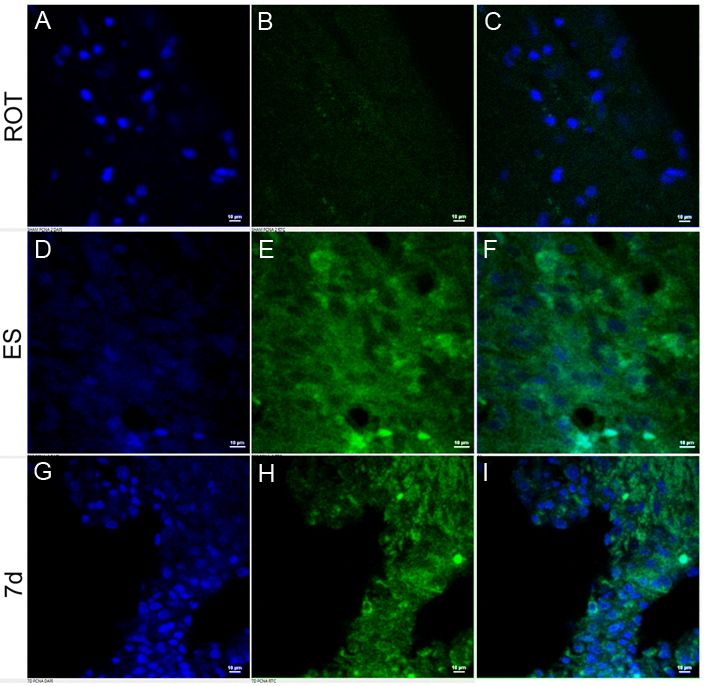

Supplement: Figure S2 — Dividing cells in the grafts. Ipasilateral striata have been processed for immune reactivity for proliferating nuclear antigen (PCNA) for the presence, if any of dividing cells. DAPI has been used to locate nuclei in the cells. Note that the middle row images that represent the undifferentiated embryonic stem cells transplanted striatum contain more PCNA positive cells than in the striatum that received 7 days differentiated cells (Lower panel). The top panel contains images from animals that were lesioned with rotenone (ROT) and received only the culture medium, without any cells. Magnification scales in figures are 10 µm. (TIF) [file pone.0072501.s002.tif]

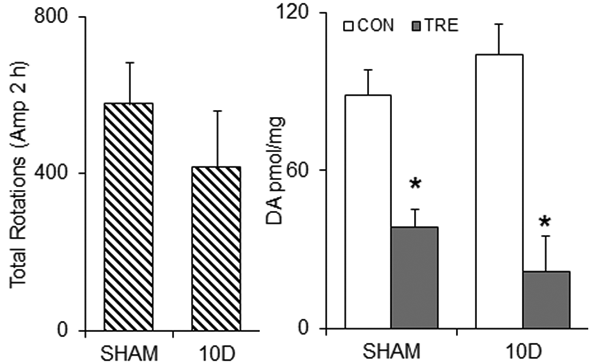

Supplement: Figure S3 — Mature neurons when transplanted do not show recovery. Ten days differentiated embryonic stem cells (10 d) were transplanted into the ipsilateral striatum of unilaterally lesioned animals. No improvement was seen in amphetamine induced rotational behaviour in the transplanted animals as compared to the sham control (A) or in the levels of the striatal dopamine levels (B). Results are presented as Mean ± SEM. *p≤0.05, n = 3. (TIF) [file pone.0072501.s003.tif]
