# Supplementary material for: Engraftment of Mouse Embryonic Stem Cells Differentiated by Default Leads to Neuroprotection, Behaviour Revival and Astrogliosis in Parkinsonian Rats
Source: PLoS One. 2013 Sep 12;8(9):e72501. doi: 10.1371/journal.pone.0072501 (PMC3772067; doi:10.1371/journal.pone.0072501)
Supplement: Table S1 — List of primers used for Semi-quantitative PCR. (DOC) [file pone.0072501.s004.doc]

**Supplementary Table S1: List of primers used for Semi-quantitative PCR**

| ***Sl. No.*** | ***Primer Name*** | ***Sequence*** |
| --- | --- | --- |
| 1. | CD11b(A) | 5’- TTACCGGACTGTGTGGAC AA-3’  5’- AGTCTCCCACCACCAAAGTG-3’ |
| 2. | CD11b(B) | 5’- AGTCCCAGTGGAACAACGAC -3’  5’-AGCACTGGGGCTAGCTGTAA -3’ |
| 3. | c-RET | 5’- GGCACCTTCTACCACTTCCA-3’  5’- CCTCCAGCACATACTTCTCC-3’ |
| 4. | ENG-1 | 5’- AGAGAGAGGGAGAGAGCGAGA-3’  5’- GGAGAGGAAGAAGGCAAAGC-3’ |
| 5. | GDNF | 5’- ACCTGGAGTTAATGTCCAACC-3’  5’- GGCATATTTGAGTCACTGCT-3’ |
| 6. | GFAP | 5’- TTGTTTGCTAGGCCCAATTC-3’  5’- CCTCGGGATCTTTTCCTTTC-3’ |
| 7. | HGPRT | 5’- CAGCGTCGTGATTAGTGATG-3’  5’- CAGCAGGTCAGCAAAGAACT-3’ |
| 8. | Iba-1 | 5’- TCGTCATCTCCCCACCTAAG-3’  5’- TCCATTGCCATTCAGATCAA-3’ |
| 9. | Lmx1b | 5’- ACTTGGGCTGTTTCTGCTG-3’  5’- GTGTGGTGAGGATGGTTCG-3’ |
| 10. | MAP2 | 5’- ACTTGGGACCTGGACGAGTA-3’  5’- AGACACAAGCCCATCCTAACA-3’ |
| 11. | Nanog | 5’- TTGGTTGGTGTCTTGCTCTTT-3’  5’- CCTTGTTCTCCTCCTCCTCA-3’ |
| 12. | Nestin | 5’- GGGAAGAGGGAGAGGAAGAA-3’  5’-AGGGCAGTTACAAGAACATTAGC-3’ |
| 13. | Nurr1 | 5’- CGGTTTCAGAAGTGCCTAGC-3’  5’- TTGCCTGGAACCTGGAATAG-3’ |
| 14. | Oct3/4 | 5’- TTCCCTCTGTTCCCGTCA-3’  5’- TGTCTACCTCCCTTGCCTTG-3’ |
| 15. | Pitx3 | 5’- AGTTTGGGCTGCTTGGTG-3’  5’- GCTGCTGGCTGGTGAAGT-3’ |
| 16. | TH | 5’- CTGTGGAGTTTGGGCTGTGT-3’  5’- CGCTGGATACGAGAGGCATA-3’ |
