# Supplementary material for: Engraftment of Mouse Embryonic Stem Cells Differentiated by Default Leads to Neuroprotection, Behaviour Revival and Astrogliosis in Parkinsonian Rats
Source: PLoS One. 2013 Sep 12;8(9):e72501. doi: 10.1371/journal.pone.0072501 (PMC3772067; doi:10.1371/journal.pone.0072501)
Supplement: Table S2 — List of antibodies and their sources used for Immuno-cytochemistry and Immuno-blotting studies. (DOC) [file pone.0072501.s005.doc]

**Supplementary Table S2:** List of antibodies and their sources used for Immuno-cytochemistry and Immuno-blotting studies

| ***Sl. No.*** | ***Name*** | ***Source*** |
| --- | --- | --- |
| 1. | NANOG | Millipore, MA, USA |
| 2. | OCT4 | Abcam, UK |
| 3. | MAP2 | Santa Cruz, CA, USA |
| 4. | TH | Abcam, UK |
| 5. | NURR1 | Abcam, UK |
| 6. | 5HT | Millipore, MA, USA |
| 7. | GFAP | Abcam, UK |
| 8. | GDNF | Abcam, UK |
| 9. | CD11b | Millipore |
| 10. | Murine Thymocyte antigen1 | Abcam, UK |
| 11. | Secondary Alexa Fluor conjugated | Invitrogen, NY, USA |
| 12. | Secondary HRP conjugated | Bangalore Genie, India |
